# Machine Learning for Automated Classification of Abnormal Lung Sounds Obtained from Public Databases: A Systematic Review

**DOI:** 10.3390/bioengineering10101155

**Published:** 2023-10-02

**Authors:** Juan P. Garcia-Mendez, Amos Lal, Svetlana Herasevich, Aysun Tekin, Yuliya Pinevich, Kirill Lipatov, Hsin-Yi Wang, Shahraz Qamar, Ivan N. Ayala, Ivan Khapov, Danielle J. Gerberi, Daniel Diedrich, Brian W. Pickering, Vitaly Herasevich

**Affiliations:** 1 Department of Anesthesiology and Perioperative Medicine, Division of Critical Care, Mayo Clinic, Rochester, MN 55905, USAypinevich@gmail.com (Y.P.); vicky8101@gmail.com (H.-Y.W.); khapovivan2021@gmail.com (I.K.); vitaly@mayo.edu (V.H.); 2 Department of Medicine, Division of Pulmonary and Critical Care Medicine, Mayo Clinic, Rochester, MN 55905, USA; 3 Department of Cardiac Anesthesiology and Intensive Care, Republican Clinical Medical Center, 223052 Minsk, Belarus; 4 Division of Pulmonary Medicine, Mayo Clinic Health Systems, Essentia Health, Duluth, MN 55805, USA; 5 Department of Anesthesiology, Taipei Veterans General Hospital, National Yang Ming Chiao Tung University, Taipei 11217, Taiwan; 6 Department of Biomedical Sciences and Engineering, National Central University, Taoyuan 320317, Taiwan; 7 Mayo Clinic Libraries, Mayo Clinic, Rochester, MN 55905, USA; gerberi.danielle@mayo.edu

**Keywords:** machine learning (ML), deep learning (DL), electronic auscultation, lung sounds, public databases

## Abstract

Pulmonary auscultation is essential for detecting abnormal lung sounds during physical assessments, but its reliability depends on the operator. Machine learning (ML) models offer an alternative by automatically classifying lung sounds. ML models require substantial data, and public databases aim to address this limitation. This systematic review compares characteristics, diagnostic accuracy, concerns, and data sources of existing models in the literature. Papers published from five major databases between 1990 and 2022 were assessed. Quality assessment was accomplished with a modified QUADAS-2 tool. The review encompassed 62 studies utilizing ML models and public-access databases for lung sound classification. Artificial neural networks (ANN) and support vector machines (SVM) were frequently employed in the ML classifiers. The accuracy ranged from 49.43% to 100% for discriminating abnormal sound types and 69.40% to 99.62% for disease class classification. Seventeen public databases were identified, with the ICBHI 2017 database being the most used (66%). The majority of studies exhibited a high risk of bias and concerns related to patient selection and reference standards. Summarizing, ML models can effectively classify abnormal lung sounds using publicly available data sources. Nevertheless, inconsistent reporting and methodologies pose limitations to advancing the field, and therefore, public databases should adhere to standardized recording and labeling procedures.

## 1. Introduction

### 1.1. Context and Objectives

Respiratory conditions are among the most common diseases associated with substantial morbidity and mortality [1], representing a growing health burden. Rapidly and reliably diagnosing pulmonary diseases is vital for establishing appropriate medical management and preventing further respiratory decompensation. Most conventional diagnostic tools (e.g., chest radiographs) can only be performed intermittently, and the standard physical exam (e.g., visual inspection and percussion) offers limited diagnostic accuracy [2,3,4]. Pulmonary auscultation is a noninvasive, safe, inexpensive, and easy-to-perform way to rapidly evaluate patients with pulmonary symptoms, making it an essential component of the clinical examination [5]. However, auscultation is operator-dependent and subject to inherent interobserver variability [2,3].

Deep learning (DL) is a subfield of machine learning (ML) and has seen increased exploration with the recent increasing computational power and large database availability [6]. In lay terms, ML allows a machine to learn rules and insights from input data, thus allowing it to apply those rules to generate predictions from data in new situations [7]. DL takes advantage of its multilayered architecture by sequentially feeding the representations into multiple layers, generating more distinguishable data points. This process allows the machine to learn highly complex functions [6].

ML and DL have shown encouraging results in healthcare when diagnosing diseases, primarily by analyzing images. For instance, radiology and pathology have benefitted from DL in disease diagnosis [8]. By utilizing large databases, classification algorithms have become increasingly accurate for detecting abnormalities in images and classifying them into multiple disease types [9], promising to reduce physician burnout and enhance test interpretations. Similarly, ML and DL can process audio signals and therefore classify sounds, such as those captured by auscultation, offering to aid clinicians in detecting and classifying heart [10] and lung [11] pathologies.

Respiratory sounds (RS) comprise relevant diagnostic information for pulmonary diseases [12]. These are heard over the chest wall and originate from the air movement in and out of the lungs during the respiratory cycle. RS interpretation in auscultation is often used in diagnosing lung pathologies, such as obstructive or restrictive respiratory diseases. As expected, these sounds are nonstationary and nonlinear, prone to noise contamination, making it hard for clinicians to detect abnormalities [13]. The diagnostic value of auscultation in detecting abnormal RSs could be improved if an objective and standardized interpretation approach is implemented [14,15]. This review aims to assess the diagnostic accuracy of ML and DL algorithms in abnormal lung sound detection and classification and evaluate the differences in methodology and reporting in the published literature to identify common issues that potentially slow down the progress of this promising field.

### 1.2. Process of Automated Abnormal Lung Sounds Classification

**Figure 1:**
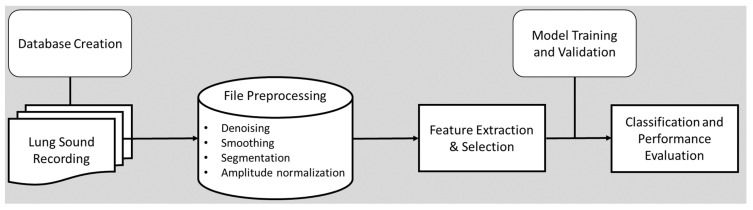
Process of automatic lung sound classification.

DL can recognize lung disorders and abnormalities based on RS analysis. These computer-assisted techniques increase the objectivity in detecting and diagnosing adventitious or pathological sounds. Figure 1 illustrates an overview of the automatic abnormal lung sounds classification process, which typically includes the following steps: audio recording, file preprocessing, feature extraction, and classification.

#### 1.2.1. Lung Sound Recording

Lung sounds are typically recorded for training healthcare workers and for research analysis; these audio samples can be broken down to objectively describe their duration, waveform, and frequency components [16]. Recordings are obtained in one of two ways, either directly by trained personnel that perform the auscultation with a device designed or adapted (with a microphone) for sound recording or by attaching sensors to the subject’s chest, which allows prolonged or continuous recording [17]. The most used sensors are piezoelectric microphones, contact microphones, electret microphones, and the more widely distributed electronic stethoscopes [11]. However, this step is subject to variability among study designs due to differences in auscultation points, recording devices, and environmental conditions.

#### 1.2.2. Audio Preprocessing

Preprocessing is an essential step, as it allows to modify the samples to better fit the purpose of the intended analysis, reduce the storage burden, and facilitate the extraction of features [18]. Among the components of preprocessing is denoising, which aims to eliminate signals that correspond to interference sources such as background noise, heartbeats, and movement [19] while preserving the valuable information; consequently, the resulting signal is cleaner and more suitable for further analysis. The most widespread denoising techniques are discrete wavelet transform (DWT), singular value decomposition (SVD), and adaptive filtering, which provide robust denoising but can be computationally expensive [20]. Smoothing is another approach, where multiple techniques are used to minimize the fluctuations in a signal, regardless of noise [21]. Other preprocessing methods include segmentation to separate breath cycles into their corresponding phases and amplitude normalization to reduce amplitude variations attributable to factors like a gain of the recording tool or subject demographics [22]. The adequate preprocessing of the audio files impacts the overall accuracy of the models [20].

#### 1.2.3. Feature Extraction

Feature extraction is identifying a set of unique properties from a signal that will be used for comparison in the classification stage. In this step, a large input signal with many redundant components can be transformed into a smaller set of representative features able to describe the original signal accurately to facilitate and expedite the classification step [23]. In general, the features are extracted from one of the following: time, frequency, and time–frequency domains [11]. Some of the established techniques for feature extraction include autoregressive models, characterized for their short training time and low variance); mel-frequency cepstral coefficients (MFCCs), which are effective for reducing dimensionality but may not capture all the nuances of complex data; and spectral and wavelet-based features, which offer multiresolution analysis and precise feature localization [11].

#### 1.2.4. Classification

ML and DL algorithms can classify the preprocessed signals and extracted features based on their characteristics, allowing them to differentiate between normal and abnormal sounds automatically. Two ways exist to feed the data into the model: holdout validation and cross-validation. In holdout validation, the dataset is divided into fixed splits of training, validation, and testing sets. The model uses training data to learn the parameters; then, the validation data allows the algorithm to search for the optimal set of hyperparameters for the model; finally, the test data is hidden during the whole model building and is used to assess the performance [24]. In the cross-validation approach, multiple partitions of the dataset are generated, allowing each partition to be used multiple times and with different purposes, potentially improving the statistical reliability of the classification results [25]. The goal of classification is to divide the sound signals into normal or abnormal [11], and more complex algorithms may go as far as differentiating between types of sounds or even underlying conditions. The performance metrics are derived from the results of this step, and measures such as accuracy or sensitivity can be calculated. Of note, the performance metrics not only depend on the used classifier but on all the previous steps.

### 1.3. Public Lung Sound Databases

The increasing popularity of artificial intelligence (AI) in biosignal classification coexists with a significant interest in developing public databases that provide the much-needed clinical data essential for developing classification models. Previous reviews have stated that biosignal databases have a clear tendency to use electrocardiogram (ECG) data [26]. Nonetheless, publicly available databases have been essential in developing abnormal lung sound [11] and cardiac [10] classification models. Undoubtedly, the interest in automatic lung sound detection has resurfaced mainly due to the widespread growth in ML and DL techniques, as well as the apparition of the mentioned publicly accessible databases [27], which narrow the gap between ML developers and available lung sound audio data. Despite the surge in the usage of large lung sound databases for DL algorithms development, a systematic evaluation has yet to examine the accuracy and reporting variations in the corresponding papers published in the last ten years.

## 2. Materials and Methods

### 2.1. Bibliographic Search

The systematic review was performed following the recommendations of the Preferred Reporting Items for Systematic Reviews and Meta-Analysis (PRISMA) statement [28]. The comprehensive literature search for articles published between January 1990 and December 2022 was carried out by an experienced specialist medical librarian (D.J.G.) on five databases, including MEDLINE, Embase, Cochrane Central Register of Controlled Trials, Web of Science, and Scopus. The full search strategy can be found in the Supplementary Files. This was confirmed by two authors independently (J.G.-M. and A.L.). The final study protocol was registered on the OSF server: https://osf.io/8sf5w.

### 2.2. Eligibility Criteria

For inclusion criteria, we defined studies that (a) proposed an ML classifier for the detection of adventitious and pathological lung sounds in adults; (b) used publicly available (online or CD) lung sounds databases; and (c) reported at least one performance metric for adequate classification, such as sensitivity, specificity, or accuracy. Book chapters, review papers, abstracts of communications or meetings, letters to the editor, commentaries to articles, unpublished works, and study protocols were excluded. Studies focused on the pediatric population or using nonpublic audio recordings were excluded. A complementary search using the references in the included papers was also conducted. Table 1 includes the detailed eligibility criteria.

### 2.3. Article Selection

Abstracts were screened by H.-Y.W. and J.G.-M. using the inclusion criteria. Full texts were independently reviewed in duplicate by eight reviewers organized in pairs (H.-Y.W., S.H., Y.P., A.T., J.G.-M., I.A., I.K., and A.L.). Disagreements were resolved during consensus meetings with a third reviewer (V.H.). Covidence software [29] was used for data collection. The studies’ outcomes were reported as the diagnostic accuracy for abnormal sound or pathology detection (sensitivity, specificity, and accuracy, when available). The types of performance measures reported depended on the approach of each study.

### 2.4. Data Extraction

The study details for the included articles were abstracted by ten independent researchers (H.-Y.W., S.H., Y.P., A.T., K.L., D.V., S.Q., J.G.-M., I.A., and I.K.) using a standardized data extraction form, and each article was assessed by two different researchers. The reviewers resolved discrepancies by consensus or in consultation with a third party, as needed. The data abstracted included the baseline details (year of publication and first author); study design (type of lung sound or pathology evaluated, DL algorithm used, feature extraction techniques, training/validation/test split, and evidence of external validation); dataset characteristics (number of recordings, auscultation points, the sensor used, and reference standard); and the performance metrics (reported as accuracy, sensitivity, and specificity).

### 2.5. Quality Assessment

We assessed the risk of bias (ROB) and applicability concerns for every included study using a modified QUADAS-2 (Quality Assessment of Diagnostic Accuracy Studies-2) instrument [30]. Ten researchers independently assessed the included articles. The quality assessment for each article was performed at least by two authors. Final adjudication and discrepancies were solved by consultation with a third author (A.L.). Given the poor standards of quality assessment (QA) reporting for AI-based diagnostic accuracy studies and the lack of validated QA tools [31], we modified the QUADAS-2 instrument to fit the purposes of this review. The four core domains for ROB evaluation were maintained, and new signaling questions tailored for this review were assessed. Given that the eligible studies used audio files from publicly available lung sound databases, such data sources were accessed when possible. This allowed for the assessment of the ROB during the audio recording creation of the database. When the corresponding lung sound database was not accessible anymore, the signaling question was answered as “N/A”, indicating a lack of information. The ROB for each domain was judged as low only when the answers to all signaling questions were “yes”; conversely, the ROB was deemed high in the presence of at least one signaling question responded to as “no”. If at least half of the signaling questions of a domain could not be assessed due to a lack of information, the ROB for the domain was deemed “unclear”. When the reference standard used to determine the sound ground truth classification was interpreted by a human or expert, this was listed as a potential source of bias and the corresponding question responded to as “no”. Applicability concerns were evaluated in the reference standard, index test, and patient selection domains, as recommended by the original QUADAS-2 instrument [32]. Notably, a significant portion of the studies used databases known to contain pediatric patients; therefore, these studies were classified as having a “high” risk regarding applicability.

## 3. Results

**Figure 2:**
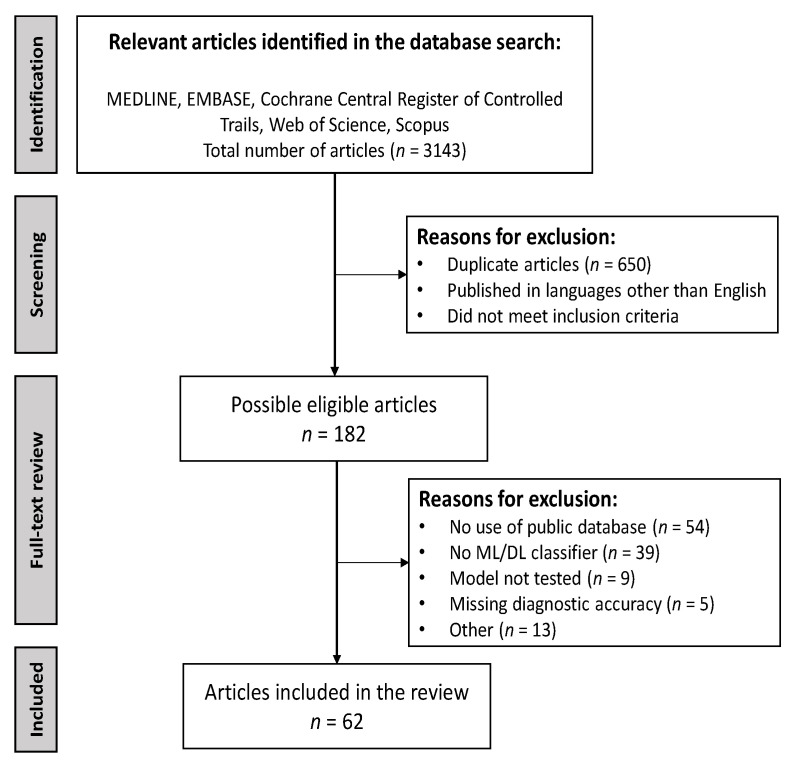
Preferred Reporting Items for Systematic Reviews and Meta-Analyses flow diagram.

A standardized approach was used for this systematic review. A database search identified a total of 3143 records. The removal of 650 duplicates left 2493 articles. Of these, 2311 articles were excluded based on title and abstract screening. From the screening, 182 full-text articles were assessed for eligibility. The main reasons for exclusion were not using audio recordings from publicly available databases and not proposing a ML/DL algorithm for abnormal lung sound classification. A few studies developed an algorithm but did not test it with patient data or lacked a performance metrics report. This study selection resulted in a total of 62 articles included in the qualitative synthesis. Figure 2 depicts this process in detail. Supplementary Table S1 presents the characteristics of each included study, namely the classifier and database used, best obtained performance metrics, and classification categories.

### 3.1. Sources of Lung Sound Recordings

As mentioned earlier, this review focuses on studies that used abnormal lung sound recordings from public databases as opposed to studies that recorded their own audio samples for the study. Creating such databases involves a series of features, including data recording protocol, recording and storage hardware, time and place of collection, and audio file labeling. Having a number of features, these biosignal repositories are prone to heterogeneity in every aspect, as well as inconsistencies, even within the same database. For this reason, the characteristics of the databases were retrieved for quality assessment, as stated in the Methods section.

**Figure 3:**
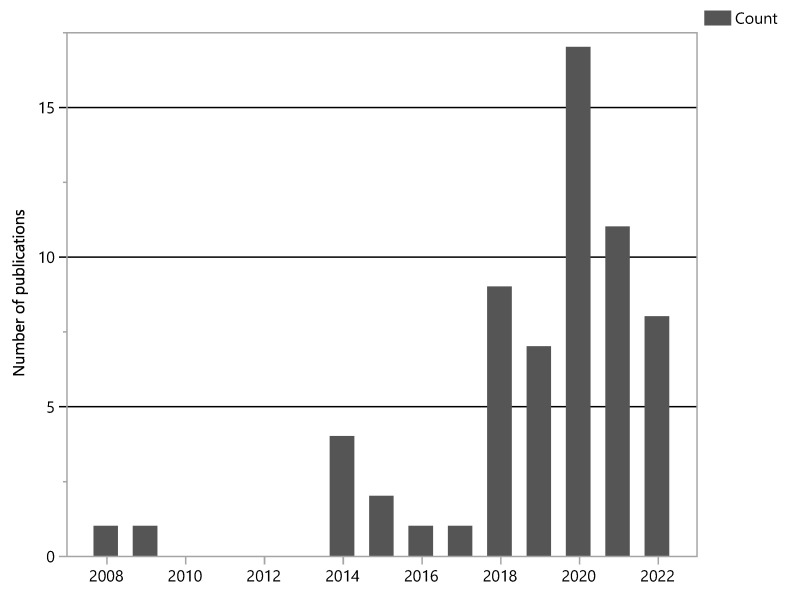
Number of included publications by year, absolute values.

As AI applications in healthcare continue to expand, the amount of available data repositories continues to grow. In this review, 17 different data sources were identified. Forty-nine articles used recordings from a single source, whereas thirteen combined audio files from multiple sources. The most frequently used online databases were the International Conference in Biomedical and Health Informatics (ICBHI) 2017 database [27] (66%) and the Respiration Acoustics Laboratory Environment (R.A.L.E.) Lung Sounds database [33] (23%), whereas other databases such as the King Abdullah University Hospital (KAUH) database or the Stethographics Lung Sound Samples were used much less often. Some studies used not currently available online databases [34,35] or only CD-accessible [36,37,38,39] databases, which prevented the quality assessment of their creation process. It is worth noting that the introduction of databases like the one by Rocha et al. [27] in 2017 led to a surge in the production of articles, as observed in Figure 3, which describes the number of studies per year of publication.

### 3.2. Features of Lung Sounds Databases

The ICBHI 2017 database contains recordings from 126 individuals, obtained by two groups of researchers using the AKG C417L Microphone (AKGC417L), 3M Littmann Classic II SE Stethoscope (LittC2SE), 3M Littmann 3200 Electronic Stethoscope (Litt3200), and Welch Allyn Meditron Master Elite Electronic Stethoscope (Meditron) at university hospitals in Portugal and Greece [27]. Respiratory experts annotated the lung sounds as “crackles, wheezes, a combination of them, or no adventitious respiratory sounds”, and the patients had conditions such as asthma, bronchiectasis, bronchiolitis, COPD, and upper and lower respiratory tract infections. As mentioned earlier, lung sounds from this database were used by most articles, as it is an open-access, readily available database that covers a wide range of diseases and abnormal sounds. In addition, the database authors suggest calculating a series of standard performance metrics, further facilitating the comparison and validation of new classification models.

The other frequently used source was the R.A.L.E. Lung Sounds database [33]. These researchers from Canada used the 3 M Littmann3200 Electronic Stethoscope (Litt3200) and Welch Allyn Meditron Master Elite Electronic Stethoscope (Meditron) to capture over 50 recordings of lung sounds, including wheezes, rhonchi, crackles, squeaks, squawks, and pleural friction rubs, annotated by respiratory experts. This database is commercially available; a license must be acquired before access. Although this resource has been available for over 20 years, a significantly smaller number of the included studies opted to use it. The license includes access to clinical cases and quizzes related to lung sounds.

Notably, one-quarter of the reported databases are only accessible via the physical acquisition of a CD-ROM [40,41,42,43,44], which impairs the quality assessment and the description of characteristics in this review. Finally, seven of all the mentioned databases were not accessible when this review was performed, in all cases due to outdated internet sources. Therefore, their characteristics could only be derived from the included articles’ descriptions in studies where combined databases were described as a whole, preventing a distinction between sources and halting their separate assessments. Further features of all the databases are described in Table 2.

### 3.3. Types of Sounds Analyzed

All eligible articles in this review targeted pulmonary sounds, but their algorithms classified sounds differently. Thirty-eight studies (61%) created algorithms that classified sounds into normal or adventitious lung sounds, with the most common ones being crackles and wheezes, although some algorithms also identified rhonchi or stridor. Twenty-one studies (34%) classified recordings into different diseases, namely with chronic obstructive pulmonary disease (COPD), asthma, pneumonia, and bronchiectasis being the most common ones. Finally, three studies (5%) created separate algorithms to distinguish adventitious lung sounds and lung pathologies.

### 3.4. Classification Models

Table 3 contains the most used classifiers in this review, a general description, and the included references corresponding to each model. As explained earlier, these techniques are the final step in the process, and they allow to classify the abnormal sounds into different categories based on the similarities and differences of their features.

Among the included manuscripts, the most used classifiers were artificial neural networks (ANN) and their subtypes and support vector machines (SVM). These techniques are examples of supervised learning algorithms, which must be trained with labeled data before classifying the unseen data points [52]. These two models can generalize appropriately these unseen data points by minimizing the risk of overfitting, resulting from having a model that learned in a way that can only apply to the training sample and poorly generalizes to unseen data [53]. Notably, many variations of ANN were tested in the included studies, ranging from the basic multilayer perceptron (MLP), composed of a series of fully connected layers [54], to the more complex recurrent neural networks (RNN) and convoluted neural networks (CNN). Ensemble methods such as Random Forests and Boosting algorithms, which combine multiple learning algorithms to improve estimates and the classification performance [55], were occasionally used in the manuscripts.

**Table 3:** The most used machine learning classification techniques.

Name		Features	Refs.
ANN	CNN RNN DNN DBN MLP	Inspired by networks of neurons, ANN models contain multiple layers of computing nodes that operate as nonlinear summing devices. These nodes communicate with each other by connection lines; the weight of each line is adjusted as the model is trained [56].	[18,35,36,38,57,58,59,60,61,62,63,64,65,66,67,68,69,70,71,72,73,74,75,76,77,78,79,80,81,82,83,84,85,86,87,88,89,90,91]
SVM		This maximal margin classifier aims to find the hyperplane in an N-dimensional space that distinctly classifies the data points [92].	[14,37,59,63,65,66,78,87,93,94,95,96,97,98,99]
k-NN		This classifier intends to classify a set of unlabeled data by assigning it to the class that contains the most similar labeled data points [100].	[14,39,59,63,65,98,99]
DT		This technique classifies data by posing questions regarding the item’s features. Each question is represented in a node, and every node directs to a series of child nodes, one for each possible answer, forming a hierarchical tree [101].	[59,87,98,102,103,104]
DA		This unsupervised learning technique intends to transform the features from a data point into a lower dimensional space, hereby maximizing the ratio of the between-class variance to the within-class variance, which results in maximized class separability [105].	[87,106,107]
RF		Random Forest is a classifier that builds multiple decision trees by using random samples of data points for each tree and random samples of the predictors; the resulting forest provides fitted values more accurate than those of a single tree [108].	[78,109]
GMM		Mixture models are derived from the idea that any distribution can be expressed as a mixture of distributions of known parameterization (such as Gaussians). Then, an optimization technique (such as expectation maximization) can be used to calculate estimates of the parameters of each component distribution [110].	[34,35,111]
HMM		The hidden Markov model creates a sequence of GMM models to explain the input data. Its main difference from GMM is that it takes account of the temporal progression of the data, whereas GMM treats each sound as a single entity [112].	[111,113,114,115]
GB		The main idea behind boosting techniques is to add a series of models into an ensemble sequentially. At each iteration, a new model is trained concerning the error of the whole ensemble [116].	[99,117]
LR		Logistic regression is a technique that describes and tests hypotheses about relationships between a categorical (outcome) variable and one or more categorical or continuous predictor variables [118].	[63,119]
NB		This supervised learning algorithm is based on the Bayes theorem. This technique works on probability distribution. The features present in the dataset are used to determine the outcome, but they are not related to other features [120].	[39]

Abbreviations: ANN: Artificial Neural Network; CNN: Convoluted Neural Network; RNN: Recurrent Neural Network; DNN: Deep Neural Network; DBN: Deep Belief Network; MLP: Multilayer Perceptron; SVM: Support Vector Machine; k-NN: k-Nearest Neighbors; DT: Decision Tree; DA: Discriminant Analysis; RF: Random Forest; GMM: Gaussian Mixture Model; HMM: Hidden Markov Model; GB: Gradient Boosting; LR: Logistic Regression; NB: Naive Bayes.

### 3.5. Performance Metrics

The evaluation of the ability of a model to adequately classify lung sounds into the appropriate category yields a series of metrics. It is of utmost importance to remember that the performance of a model not only depends on the ML/DL classifier but also on all the steps that precede it (audio recording, preprocessing, feature selection, and model training). These metrics are helpful when comparing different models that use the same data sources but, understandably, are not a reliable way to compare models across different databases. Some databases, like the ICBHI 2017 Challenge [27], suggest that researchers use specific performance metrics to evaluate their models; nonetheless, for this review, the evaluated performance metrics were accuracy and/or sensitivity and specificity. The accuracy for classification into abnormal sound categories ranged between 49.43 [102] and 100.00 [18]. Meanwhile, the sensitivity and specificity ranged between 17.80 [90] and 100.00 [18,65] and 59.69 [113] and 100.00 [38,64], respectively. On the other hand, the lowest and highest accuracies for models that classified sounds into disease classes were 69.40 [99] and 99.62 [69]. For the same studies, the sensitivity ranged between 28.00 [77] and 100.00 [63], whereas the specificity ranged between 81.00 [77] and 100.00 [88]. Remarkably, the reported metrics were highly heterogeneous between studies, limiting direct comparisons.

### 3.6. Quality Assessment

**Figure 4:**
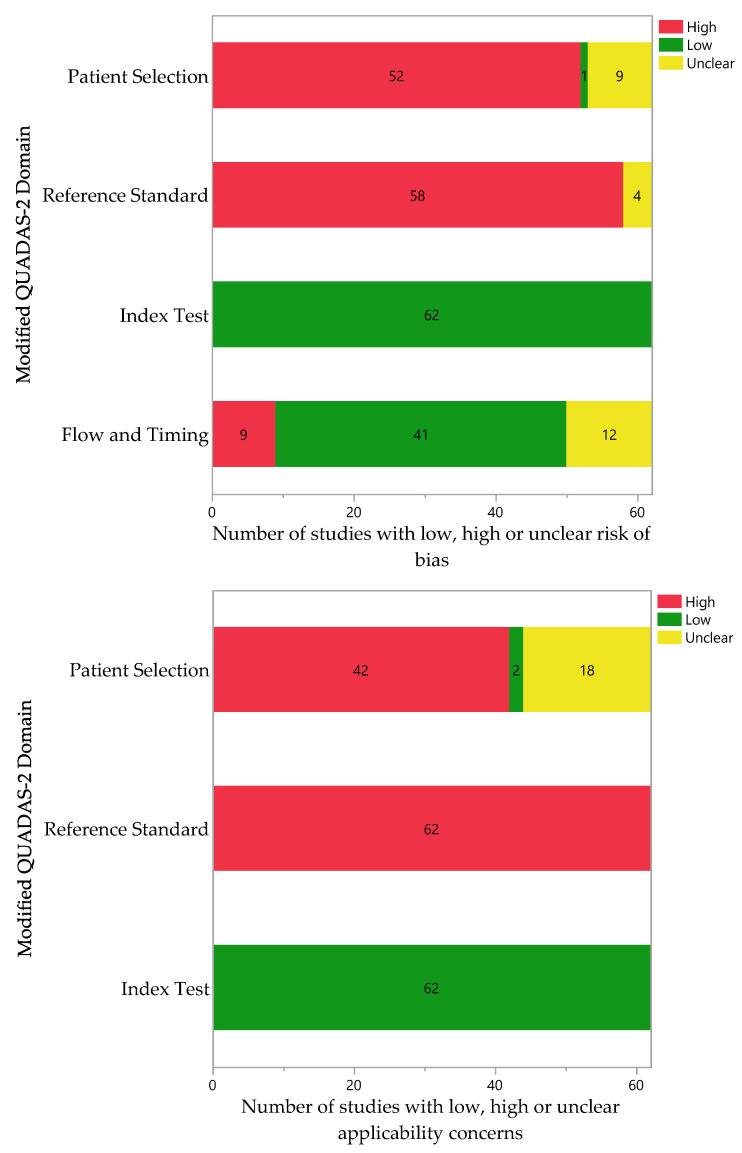
Quality assessment summary plots for the risk of bias (**top**) and applicability concerns (**bottom**). Presented as the number of articles with high, unclear, or low risk/concerns across each domain of the modified QUADAS-2 tool. (Green: low risk of bias; red: high risk of bias; yellow: unclear risk of bias).

Given the lack of a validated tool for the quality assessment of diagnostic studies that use artificial intelligence, we optimized a version of the QUADAS-2 tool to evaluate the risk of bias and applicability concerns. After using this tool, all the studies were classified as having an overall high ROB, with most concerns over the patient selection and the reference standards. The high ROB in these domains directly relates to using public databases to obtain audio files. These sources often do not follow a specific sound recording protocol, use multiple devices, and rely on interpretation by an individual to assign labels to each recording. In addition, the characteristics of each database are rarely available, further halting the quality assessment process. None of the included studies had concerns regarding applicability in the index test domain, while almost all the studies had serious or unclear concerns in the patient selection and reference standard domains. The concern arose due to the poor description of the patient population in the included papers and/or data sources, which creates a risk of including pediatric patients, for example. Also, using expert annotation as a reference standard precludes the reliability of the labels for each study, raising concerns in this domain. Tables S2 and S3 in the Supplementary Files contain the individual assessment results of the risk of bias and applicability concerns, respectively. Figure 4 summarizes the quality assessment findings.

## 4. Discussion

Our systematic review provides a comprehensive update on using contemporary ML and DL models. To the best of our knowledge, this work offers a much-needed update that highlights the advances in automatic lung sound classification during the last six years, focusing on the introduction of large public databases that have encouraged further research in the field. The apparition of large public data sources in recent years has led to an increasing number of studies to share their lung sound audio samples, ideally facilitating comparisons between models. Nonetheless, a detailed description of the databases and studies is necessary to identify the emerging issues in the field and the progress made so far. Supplementary Table S1 highlights the models identified in our systematic review with the best accuracy, sensitivity, and specificity performance metrics.

### 4.1. Clinical and Scientific Relevance

Machine learning (ML) and deep learning (DL) techniques are of increasing importance and great functionality in the identification and classification of normal and abnormal lung sounds [121], although, historically, a bedside clinician has been the key decider for identifying and classifying various normal and abnormal lung sounds, such as vesicular lung sounds, crackles, and wheezes. This information carries various degrees of diagnostic certainty, depending on the experience level and skill set. The inability to identify and accurately classify lung sounds could significantly impact the delay in diagnosis and downstream management [122]. Güler et al. described the initial work of utilizing a neural networks-genetic algorithm approach to advance the field in the lung sounds classification [123]. Additionally, they employed a multilayer perception neural network employing a backpropagation training algorithm to predict normal or abnormal lung sounds (such as crackles or wheezes), ultimately yielding a model with promising performance, with correct classification rates of up to 93% for all lung sounds. Early studies like the aforementioned served as the groundwork for future authors that intended to improve the methodology and capabilities of their models.

The traditional methods of lung sound analysis depend heavily on the expertise of bedside clinician, which has a significant subjectivity. Their results could be prone to interobserver variability, and the same observer could potentially classify the same lung sounds differently. ML and DL algorithms could minimize that variability and could provide objectivity, offering several advantages. In addition to this, the ML and DL methods could extract the relevant features from lung sound recordings, capturing characteristics that were not picked up by pulmonary auscultation [124,125], such as the frequency content, temporal patterns, and spectral properties, to name a few. These additional characteristics could further enrich a training dataset’s diversity and variability, enabling accurate classification and identification for future studies.

With the technical advances in computing, machine learning in deep planning models such as support vector machines (SVM), Random Forests, and neural networks have been utilized at an increasing pace to label and classify lung sound data [126]. The increasing fidelity and improvement in the performance of the resulting models could provide accurate diagnostic and predictive enrichment for specific disease states, such as pneumonia, pleural effusions, consolidations, and airway diseases (rhonchi and wheezing), among others.

Deep learning models such as neural networks (NNs) could provide the benefit of real-time monitoring of lung sounds. If developed and validated clinically, these models could be used for real-time lung sound monitoring in acute care settings (such as hospitals) and remote monitoring environments such as nursing homes, rehabilitation facilities, or even at home [119,127]. The real-time analysis could allow for the early detection of disease states, enabling an actionable point of timely intervention and overall improvement in healthcare delivery. Potential challenges that could be anticipated include difficulty in noise reduction, thereby impeding the signal-to-noise ratio and diluting the diagnostic information present in the audio signals. With the advent of precision and personalized medicine, these machine learning and deep planning models can be trained on high-quality datasets with high signal-to-noise ratios, thereby allowing the further design of personalized models that could consider individual variations in lung sounds, accounting for age, sex, body habitus, disease progression, ethnicity, and other factors contributing to patient-to-patient variability [128,129,130].

### 4.2. Opportunities and Barriers

Utilizing machine learning and deep learning techniques in this realm has several strengths and advantages. ML and DL algorithms will enable the automated analysis of lung sounds, thereby relying less on human subjective nature and interpretation. This automation will improve efficiency with a reduction in interobserver variability. ML and DL models also excel in recognizing complex patterns in data that are either unknown or difficult to recognize by humans; this concept also holds true in lung sound identification [131,132]. As highlighted above, one of the biggest advantages will be the real-time monitoring of patients’ lung sounds remotely in a hospital setting and their community (at home). This will facilitate the early detection of physiological abnormality, and we will provide an actionable point of timely intervention. Adaptability and self-limiting from new data will allow for continuous improvement in performance and fidelity over time. Despite all the advantages highlighted above, these ML and DL models have inherent weaknesses. The availability of high-quality and labeled lung sound datasets can be a challenge, as highlighted by many manuscripts included in our systematic review. Heterogeneity in the database creation process inevitably leads to a scenario where comparisons between models are not possible. Stakeholder engagement for creating well-annotated datasets with patient populations can be time-consuming and expensive. Databases lacking in diversity could affect the generalizability and potentially increase healthcare disparities in diagnostics and healthcare delivery. Physiologically, lung sounds could vary significantly due to various patient factors such as body habitus, body position, patient movement, disease timeline, and recording conditions. This variability in lung sound recording could present hurdles in realizing consistent and accurate classification if not accurately annotated.

### 4.3. Strengths and Limitations

The strengths of this review include the extensive literature search, as well as the individual evaluations and detailed descriptions of the data sources. Furthermore, we developed a new approach to the quality assessment of the included articles, given the lack of validated assessment tools for diagnostic accuracy studies that use artificial intelligence. Our study was limited by the impossibility to perform a meta-analysis, given the heterogeneity in the performance reporting and data sources. Similarly, we could not access a large portion of the older databases, preventing us from evaluating and describing their characteristics. Notably, our review focused on studies in English that used public databases as their source of audio samples, excluding those published in other languages and those that opted for a different approach, such as collecting their own sounds. Although omitted in our work, these studies may provide valuable contributions to the development of the field.

### 4.4. Future Work

As noted, while the machine learning and deep learning techniques have, so far, offered valuable strengths in the accurate identification and classification of lung sounds, improved efficiency, and provided the possibility of real-time remote monitoring, they also face certain limitations. To harness the full potential of these techniques in healthcare, we need to overcome the challenges surrounding data availability, data security, accurate labeling and interpretation, and domain expertise. As evidenced by the results of this review, public databases are an essential component in the progress of the field of automatic lung sound classification, but researchers interested in developing their own database should aim to create a standardized approach to the recording, storage, and share processes, which will ultimately lead to more reliable comparisons between models. Utilizing ML and DL techniques for lung sound analysis could raise ethical concerns regarding patient privacy, data security, and other regulatory oversight needs [133]. Therefore, these concerns should be clearly addressed when developing public databases.

## 5. Conclusions

In conclusion, we see a rising trend of more ML and DL techniques demonstrating promise in appropriate identification and classification, increasing the accuracy for various lung sound characteristics. Automating the analysis process and enriching the currently publicly available databases could offer a precious source of objective and accurate diagnostic utility. With further advancements in computational prowess, these techniques have the potential to provide better-personalized precision medicine and accurate assessments of respiratory conditions, aiding in diagnosis, monitoring, and treatment.

## Figures and Tables

**Table 1:** Population, Intervention, Comparator, Outcome, and Study Design (PICOS) eligibility criteria for the systematic review.

Parameter	Inclusion Criteria	Exclusion Criteria
Population	Total or majority of adult (age ≥ 17) cohort. Underlying pulmonary disease, causing abnormal respiratory sounds. Audio files obtained from a publicly available database. Manuscripts published in English.	Studies focusing on pediatric cohorts. Focus on cardiac auscultation sounds. Audio files obtained from private databases. Audio files self-collected for the purpose of the study. Manuscripts published in languages other than English.
Intervention	Use of at least one machine learning algorithm to classify abnormal respiratory sounds.	No machine learning algorithm used.
Comparator	Labeling of abnormal sounds provided by the source database.	No labeling provided by the source database.
Outcomes	Report of at least one of the following performance metrics: accuracy, sensitivity, and specificity.	No performance metric report.
Study Designs	Machine learning algorithm development, comparison, validation, and hyperparameter tuning.	Book chapters. Reviews. Abstracts. Letters to the editor. Unpublished work. Study protocol.

**Table 2:** Abnormal lung sounds sources are mentioned in the included articles. Some databases are no longer accessible or their characteristics are not described. (Contents are sorted by availability, last column, and country of origin, second column).

Database or Author Name	Country	Participants Number (Total (M/F); HC)	Abnormal Lung Sounds Labeled	Pathologies Labeled	Availability ^1^	Ref.
R.A.L.E. Lung Sounds 3.2	Canada	70 (-); 17	Crackles, Wheezes, Squawk, Stridor, Rhonchi	Asthma, COPD, Bronchiolitis, Laryngeal web, Bronchogenic carcinoma, Lung fibrosis, Cystic fibrosis.	Available online	[33]
ICBHI 2017 Challenge Database	Greece, Portugal	126 (46/79); 26	Crackles, Wheezes, Crackles + Wheezes	Asthma, Bronchiectasis, Bronchiolitis, COPD, Pneumonia, LRTI, URTI	Available online	[27]
KAUH database	Jordan	120 (43/69); 35	Crackles, Wheezes, Crepitations, Bronchial sounds, Crackles + Wheezes, Crackles + Bronchial	Asthma, Pneumonia, COPD, Bronchitis, Heart failure, Lung fibrosis, Pleural effusion	Available online	[45]
RespiratoryDatabase@TR	Turkey	77 (64/13); 30	Crackles, Wheezes	Asthma, COPD	Available online	[46]
Thinklabs Lung Sounds Library	United States	-	Crackles, Wheezes, Pleural rub, Rhonchi, Stridor	Asthma, Bronchiolitis, COPD, Laryngomalacia, Pulmonary edema	Available online	[47]
East Tennessee State University Pulmonary Breath Sounds	United States	-	Crackles, Pleural rub, Stridor, Wheezing, Rhonchus	-	Available online	[48]
ASTRA database	France	-	-	-	CD-ROM	[40]
Auscultation Skills: Breath & Heart Sounds	United States	-	-	-	CD-ROM	[41]
Fundamentals of Lung and Heart Sounds	United States	-	-	-	CD-ROM	[42]
Heart and Lung Sounds Reference Library, Wrigley	United States	-	Bronchial, Bronchovesicular, Rhonchi, Pneumonia, Wheezes, Bronchophony, Crackles, Stridor,	-	CD-ROM	[43]
Understanding Lung Sounds, Lehrer	United States	-	Crackles, Wheezes	-	CD-ROM	[44]
Bahoura 1999	France	-	-	-	Undefined	[49]
Hsiao 2020	Taiwan	22 (12/10); -	Crackles, Wheezes	-	Undefined	[50]
Bogazici University Lung Acoustics Laboratory	Turkey	-	-	Bronchiectasis, Interstitial lung disease	Undefined	-
CORA database	Ukraine	-	-	Bronchitis, COPD	Undefined	[51]
Stethographics Lung Sound Samples ^2^	United States	-	-	-	Undefined	-
3M Littmann Lung Sounds Library	United States	-	-	-	Undefined	-
Mediscuss Respiratory Sounds ^2^	-	-	-	-	Undefined	-

Abbreviations: M: Males; F: Females; HC: Healthy Controls; COPD: Chronic Obstructive Pulmonary Disease; LRTI: Lower Respiratory Tract Infection; URTI: Upper Respiratory Tract Infection. ETSU: East Tennessee State University; ICBHI: International Conference on Biomedical and Health Informatics; KAUH: King Abdullah University Hospital; R.A.L.E: Respiratory Acoustics Laboratory Environment. ^1^ Availability at the time of submission. ^2^ This database was mentioned in one of the included articles but could not be found in this review.

## Data Availability

Not applicable.

## Supplementary Materials

The following supporting information can be downloaded at: https://www.mdpi.com/article/10.3390/bioengineering10101155/s1, Table S1: List of the included studies’ main characteristics. Table S2: Individual risk of bias assessments. Table S3: Individual applicability concerns assessments. Database search strategy.
