# Supplementary material for: Machine Learning for Automated Classification of Abnormal Lung Sounds Obtained from Public Databases: A Systematic Review
Source: Bioengineering (Basel). 2023 Oct 2;10(10):1155. doi: 10.3390/bioengineering10101155 (PMC10604310; doi:10.3390/bioengineering10101155)
Supplement: Supplementary file 1 [file bioengineering-10-01155-s001.zip › bioengineering-2577716-supplementary.pdf]

bioengineering

Supplementary Materials

Machine Learning for Automated Classification of Abnormal Lung Sounds Obtained from Public Databases: A Systematic Review

Juan P. Garcia-Mendez [1], Amos Lal [2,*], Svetlana Herasevich [1], Aysun Tekin [1], Yuliya Pinevich [1,3], Kirill Lipatov [4], Hsin-Yi Wang [1,5,6], Shahraz Qamar [1], Ivan N. Ayala [1], Ivan Khapov [1], Danielle J. Gerberi [7], Daniel Diedrich [1], Brian W. Pickering [1] and Vitaly Herasevich [1]

[1] Department of Anesthesiology and Perioperative Medicine, Division of Critical Care, Mayo Clinic, Rochester, MN 55905, USA; ypinevich@gmail.com (Y.P.); vicky8101@gmail.com (H.-Y.W.); khapovivan2021@gmail.com (I.K.); vitaly@mayo.edu (V.H.)
[2] Department of Medicine, Division of Pulmonary and Critical Care Medicine, Mayo Clinic, Rochester, MN 55905, USA
[3] Department of Cardiac Anesthesiology and Intensive Care, Republican Clinical Medical Center, 223052 Minsk, Belarus
[4] Division of Pulmonary Medicine, Mayo Clinic Health Systems, Essentia Health, Duluth, MN 55805, USA
[5] Department of Anesthesiology, Taipei Veterans General Hospital, National Yang Ming Chiao Tung University, Taipei 11217, Taiwan
[6] Department of Biomedical Sciences and Engineering, National Central University, Taoyuan 320317, Taiwan
[7] Mayo Clinic Libraries, Mayo Clinic, Rochester, MN 55905, USA; gerberi.danielle@mayo.edu
* Correspondence: lal.amos@mayo.edu

Table S1. List of included studies main characteristics. Sorted by author.

Author, Year	Classifier (method)	Feature Extraction (method)	Database(s)	Performance Metrics	Categories	Classification Classes
Acharya, 2020 [57]	CNN + RNN(Bi-LSTM)	Mel-frequency Spectrogram	ICBHI 2017	Accuracy: * Sens: 48.63 Spec: 84.14	Adventitious sounds	Normal vs Wheeze vs Crackle vs Crackle and Wheeze
Alqudah, 2022 [58]	CNN + RNN(LSTM)	Convolutional Neural Networks	(1) ICBHI 2017, (2) KAUH database	Non-augmented dataset: Accuracy: 99.37 Sens: 98.5 Spec: 99.87 Augmented dataset: Accuracy: 99.53 Sens: 98.80 Spec: 99.92	Diseases	Healthy vs Asthma vs Fibrosis vs COPD vs BRON vs HF vs HF+COPD vs HF + Fibrosis vs Pleural effusion vs Pneumonia
Altan, 2020 [59]	k-NN, DT, SVM, DBN	Hilbert-Huang Transform	RespiratoryDatabase@TR	k-NN: Accuracy: 83.5 Sens: 79.33 Spec: 87.67 DT: Accuracy: 84.83 Sens: 87.67	Diseases	Healthy vs COPD

				Spec: 82.00<br><br>SVM:<br>Accuracy: 89.62<br>Sens: 90.67<br>Spec: 88.59<br><br>DBN:<br>Accuracy: 93.67<br>Sens: 91.00<br>Spec: 96.33		
Bahoura, 2009 [35]	GMM, MLP	Fourier Transform; Linear Predictive Coding; Cepstral Analysis; Wavelet Transform	(1) Bahoura 1999 (2) R.A.L.E. Lung Sounds, (3) ASTRA database, and (4) Others (non-specified)	GMM:<br>Accuracy: *<br>Sens: 97.20<br>Spec: 94.20	Adventitious sounds	Normal vs Wheeze
Bahoura, 2018 [60]	MLP	Mel-Frequency Cepstral Coefficients	(1) Bahoura 1999 (2) R.A.L.E. Lung Sounds, (3) ASTRA database, and (4) Others (non-specified)	XSG:<br>Accuracy: 86.05<br>Sens: 81.52<br>Spec: 88.51<br><br>MATLAB:<br>Accuracy: 86.07<br>Sens: 81.71<br>Spec: 88.43	Adventitious sounds	Normal vs Wheeze
Bardou, 2018 [61]	CNN (Ensemble model)	Mel-Frequency Cepstral Coefficients	R.A.L.E. Lung Sounds	Augmented + ensemble:<br>Accuracy: 95.56<br>Sens: *<br>Spec: *	Adventitious sounds	Normal vs Coarse crackle vs Fine crackle vs Monophonic wheeze vs Polyphonic wheeze vs Squawk vs Stridor
Basu, 2020 [62]	NN	Mel-Frequency Cepstral Coefficients	ICBHI 2017	Accuracy: 95.67<br>Sens: 95.65<br>Spec: *	Diseases	Healthy vs Bronchiolitis vs Bronchiectasis vs Pneumonia vs URTI vs COPD
Boujelben, 2018 [93]	SVM	Mel-Frequency Cepstral Coefficients	(1) Bahoura 1999 (2) R.A.L.E. Lung Sounds, (3) ASTRA database, and (4) Others (non-specified)	Default XSG:<br>Accuracy: 93.72<br>Sens: 89.72<br>Spec: 96.06<br><br>Optimized XSG:<br>Accuracy: 93.49<br>Sens: 89.36<br>Spec: 96.06<br><br>MATLAB:<br>Accuracy: 93.59<br>Sens: 89.72<br>Spec: 96.06	Adventitious sounds	Normal vs Wheeze

Brunese, 2022 [63]	k-NN, SVM, NN, LR	Mel-Frequency Cepstral Coefficients; Root Mean Square; Chromagram; Spectral Centroid; Spectral Roll-off; Zero Crossing Rate	ICBHI 2017	Task 1:<br><br>k-NN:<br>Accuracy: *<br>Sens: 99.7<br>Spec: 96.5<br><br>SVM:<br>Accuracy: *<br>Sens: 100<br>Spec: 96.6<br><br>NN:<br>Accuracy: *<br>Sens: 98.8<br>Spec: 97.9<br><br>LR:<br>Accuracy: *<br>Sens: 98.2<br>Spec: 97.6<br><br>Task 2:<br><br>k-NN:<br>Accuracy: *<br>Sens: 90.80<br>Spec: 88.30<br><br>SVM:<br>Accuracy: *<br>Sens: 90.70<br>Spec: 89.00<br><br>NN:<br>Accuracy: *<br>Sens: 93.10<br>Spec: 91.70<br><br>LR:<br>Accuracy: *<br>Sens: 90.40<br>Spec: 88.60	Diseases	Task 1: Healthy vs non-Healthy<br><br>Task 2: Asthma vs Bronchiectasis vs Bronchiolitis vs COPD vs Pneumonia vs LRTI vs URTI
Chen, 2019 [64]	CNN (Residual Network)	Optimized S-Transform	ICBHI 2017	OST (Optimized S control)-ResNet:<br>Accuracy: 98.79<br>Sens: 96.27<br>Spec: 100	Adventitious sounds	Normal vs Wheeze vs Crackle vs Wheeze and Crackle

				ST-ResNet:<br>Accuracy: 97.79<br>Sens: 93.17<br>Spec: 100<br><br>STFT-ResNet:<br>Accuracy: 93.98<br>Sens: 86.96<br>Spec: 97.33		
Chen, 2019 [65]	SVM, ELM, k-NN	Enhanced Generalized S-Transform	(1) ICBHI 2017, (2) R.A.L.E. Lung Sounds	Accuracy: 99.52<br>Sens: 100<br>Spec: 99.27	Adventitious sounds	Normal vs Wheeze
Datta, 2017 [37]	SVM	Welch Method (Spectral Features); Spectrogram Features; Discrete Wavelet Transform; Mel-Frequency Cepstral Coefficients	(1) R.A.L.E. Lung Sounds, (2) Steven Lehrer Sound Repository (CD) (3) Healthy sounds were self-recorded	Overlapping:<br>Accuracy: 85.00<br>Sens: *<br>Spec: *<br><br>Non-overlapping:<br>Accuracy: 80.00<br>Sens: *<br>Spec: *	Adventitious sounds	Normal vs Abnormal (Wheeze, Crackle, Squawks, Stridor, Grunt, Squeak, Pleural rub)
Demir, 2020 [67]	CNN (LDA + RSE)	Convolutional Neural Networks	ICBHI 2017	Accuracy: 71.15<br>Sens: 61.00<br>Spec: 86.00	Adventitious sounds	Normal vs Wheeze vs Crackle vs Crackle and Wheeze
Demir, 2020 [66]	CNN, SVM	Short-time Fourier Transform	ICBHI 2017	Deep feature + SVM:<br>Accuracy: 65.50<br>Sens: 53.00<br>Spec: 83.00<br><br>Transfer learning + Softmax:<br>Accuracy: 63.09<br>Sens: *<br>Spec: *	Adventitious sounds	Normal vs Wheeze vs Crackle vs Crackle and Wheeze
D. Perna, 2018 [68]	CNN	Mel-Frequency Cepstral Coefficients	ICBHI 2017	Task 1:<br>Accuracy: 83.00<br>Sens: *<br>Spec: *<br><br>Task 2:<br>Accuracy: 82.00<br>Sens: *<br>Spec: *	Diseases	Task 1: Healthy vs Unhealthy<br><br>Task 2: Healthy vs Chronic disease vs non-Chronic disease

Fraiwan, 2022 [69]	CNN + RNN (BDLSTM)	Convolutional Neural Networks	(1) ICBHI 2017 (only included 110 patients), (2) KAUH database	CNN only: Accuracy: 99.04 Sens: 93.02 Spec: 99.33<br><br>BDLSTM only: Accuracy: 98.16 Sens: 90.06 Spec: 98.61<br><br>CNN + RNN (BDLSTM): Accuracy: 99.62 Sens: 98.43 Spec: 99.69	Diseases	Normal vs Asthma vs Pneumonia vs Bronchiectasis vs COPD vs Heart failure
Gairola, 2017 [70]	CNN	Mel-frequency Spectrogram	ICBHI 2017	Task 1: Accuracy: 77.00 Sens: 73.10 Spec: 80.90<br><br>Task 2: Accuracy: 68.50 Sens: 53.70 Spec: 83.30	Adventitious sounds	Task 1: Normal vs Abnormal<br><br>Task 2: Normal vs Wheeze vs Crackle vs Crackle and Wheeze
Garcia-Ordas, 2020 [71]	CNN	Mel-frequency Spectrogram	ICBHI 2017	Task 1: Accuracy: * Sens: 98.80 Spec: 98.60<br><br>Task 2: Accuracy: * Sens: 98.50 Spec: 99.00	Diseases	Task 1: Healthy vs Chronic disease vs non-Chronic disease<br><br>Task 2: Healthy vs Bronchiolitis vs Bronchiectasis vs Pneumonia vs URTI vs COPD
G. Chambres, 2018 [102]	Boosted DT	Mel-Frequency Cepstral Coefficients	ICBHI 2017	Accuracy: 49.43 Sens: 20.81 Spec: 78.05	Adventitious sounds	Normal vs Wheeze vs Crackle vs Crackle and Wheeze
Hazra, 2020 [72]	CNN	Mel-Frequency Cepstral Coefficients	ICBHI 2017	Accuracy: 92.39 Sens: 50.83 Spec: *	Diseases	Healthy vs Bronchiolitis vs Bronchiectasis vs Pneumonia vs URTI vs COPD
Jaber, 2020 [109]	RF	Mel-Frequency Cepstral Coefficients	R.A.L.E. Lung Sounds	Accuracy: 99.04 Sens: 98.70 Spec: 99.49	Adventitious sounds	Normal vs Coarse crackle vs Fine crackle vs Monophonic wheeze vs Polyphonic wheeze vs Squawk vs Stridor
Jakovljevic, 2018 [113]	HMM	Mel-Frequency Cepstral Coefficients	ICBHI 2017	Accuracy: * Sens: 42.32 Spec: 59.69	Adventitious sounds	Normal vs Wheeze vs Crackle vs Crackle and Wheeze

Jung, 2021 [73]	DS-CNN	Mel-Frequency Cepstral Coefficients; Short-time Fourie Transform	Hsiao 2020	Accuracy: 85.74<br>Sens: 86.25<br>Spec: 86.75	Adventitious sounds	Normal vs Wheeze vs Crackle vs Unknown
Kochetov, 2018 [74]	NM-RNN	Mel-Frequency Cepstral Coefficients	ICBHI 2017	Accuracy: *<br>Sens: 58.40<br>Spec: 73.00	Adventitious sounds	Normal vs Wheeze vs Crackle vs Crackle and Wheeze
Kok, 2019 [103]	DT	Mel-Frequency Cepstral Coefficients; Discrete Wavelet Transform; Time Domain Features	ICBHI 2017	Accuracy: 87.1<br>Sens: 86.80<br>Spec: 93.60	Adventitious sounds	Task 1: Normal vs abnormal<br><br>Task 2: Normal vs Wheeze vs Crackle vs Crackle and Wheeze
Li, 2022 [75]	Explainable CNN (based on fuzzy DT)	Mel-frequency Spectrogram	ICBHI 2017	Task 1:<br>Accuracy: *<br>Sens: 61.00<br>Spec: 82.00<br><br>Task 2:<br>Accuracy: *<br>Sens: 98.00<br>Spec: 92.00	Both adventitious sounds and diseases	Task 1: Crackle vs Normal vs Wheeze<br>Task 2: COPD vs Healthy vs Pneumonia
Li, 2021 [76]	CNN (ResNet+Attention-augmented convolution)	Dual tunable Q-factor Wavelet Transform; Short-time Fourier Transform	ICBHI 2017	Accuracy: *<br>Sens: 36.36<br>Spec: 71.44	Adventitious sounds	Normal vs Wheeze vs Crackle vs Crackle and Wheeze
Lu, 2008 [34]	GMM	Wavelet Packet Transform	(1) ASTRA database, (2) R.A.L.E. Lung Sounds	Accuracy: *<br>Sens: 92.90<br>Spec: 94.40	Adventitious sounds	Normal vs Crackle vs Wheeze
Minami, 2019 [77]	CNN	Short-Time Fourier Transform; Wavelet Transform	ICBHI 2017	Accuracy: *<br>Sens: 28.00<br>Spec: 81.00	Diseases	Bronchiectasis vs pneumonia vs bronchiolitis vs COPD vs URTI vs normal
Monaco, 2020 [78]	MLP, SVM, DNN, RF	Multi-time Scale Feature Extraction	ICBHI 2017	MLP:<br>Accuracy: 85.00<br>Sens: *<br>Spec: *	Adventitious sounds	Normal vs Crackle vs Wheeze vs Crackle and Wheeze

				RF:<br>Accuracy: 84.00<br>Sens: *<br>Spec: *<br><br>DNN:<br>Accuracy: 82.00<br>Sens: *<br>Spec: *<br><br>SVM:<br>Accuracy: 81.00<br>Sens: *<br>Spec: *		
Mukherjee, 2021 [79]	MLP	Linear Predictive Cepstral Coefficient	ICBHI 2017	Accuracy: 99.22<br>Sens: 99.17<br>Spec: 99.26	Diseases	Healthy vs non-Healthy
Naqvi, 2020 [106]	QDA	Mel-Frequency Cepstral Coefficients; Gammatone Cepstral Coefficients; Time Domain Features; Spectral Domain Features	ICBHI 2017	Accuracy: 99.80<br>Sens: *<br>Spec: *	Diseases	Healthy, COPD, Pneumonia
Naves, 2016 [39]	k-NN, Naive Bayes	Higher-order Statistics	Steven Lehrer Sound Repository 2002 (CD)	Accuracy: 94.60<br>Sens: *<br>Spec: *	Adventitious sounds	Normal vs coarse crackle vs Fine crackle vs Monophonic wheeze vs Polyphonic wheeze
Ngo, 2021 [80]	C-DNN + autoencoder	Short-time Fourier Transform, Gammatone	ICBHI 2017	Accuracy: *<br>Sens: 30.00<br>Spec: 69.00	Adventitious sounds	Normal vs Crackle vs Wheeze vs Crackle and Wheeze
Nguyen, 2020 [81]	CNN, SE	Fast Fourier Transform	ICBHI 2017	Task 1:<br><br>CNN:<br>Accuracy: *<br>Sens: 69.10<br>Spec: 86.10<br><br>SE:<br>Accuracy: *<br>Sens: 69.40<br>Spec: 87.30<br><br>Task 2:	Adventitious sounds	Task 1: Normal vs Crackle vs Wheeze vs Crackle and Wheeze<br><br>Task 2: Normal vs Abnormal

				CNN:<br>Accuracy: *<br>Sens: 80.40<br>Spec: 86.10<br><br>SE:<br>Accuracy: *<br>Sens: 80.10<br>Spec: 87.30		
Ntalampiras, 2020 [114]	DAG-HMM	Discrete Wavelet Transform	ICBHI 2017	Accuracy: 50.10<br>Sens: *<br>Spec: *	Adventitious sounds	Normal vs Crackle vs Wheeze vs Crackle and Wheeze
Ntalampiras, 2020 [111]	HMM + GMM	Mel-Frequency Cepstral Coefficients; Discrete Wavelet Transform	ICBHI 2017	Accuracy: 66.70<br>Sens: *<br>Spec: *	Adventitious sounds	Normal vs Abnormal
Oletic, 2014 [104]	DT	Short-time Fourier Transform	(1) R.A.L.E. Lung Sounds, (2) Stethographics Lung Sound Samples, (3) Mediscuss Respiratory Sounds, (4) 3M Littmann Lung Soundsw Library, (5) East Tennessee State University Breath Sounds	Accuracy: 92.96<br>Sens: 96.92<br>Spec: 91.21	Adventitious sounds	Normal vs Wheeze
Oletic, 2018 [115]	HMM	CS-recovered Short-time Fourier Transform	(1) R.A.L.E. Lung Sounds, (2) Stethographics Lung Sound Samples, (3) Mediscuss Respiratory Sounds, (4) 3M Littmann Lung Soundsw Library, (5) East Tennessee State University Breath Sounds	Accuracy: 94.91<br>Sens: 89.34<br>Spec: 96.28	Adventitious sounds	Normal vs Wheeze
Oweis, 2015 [38]	ANN, ANFIS	Fast Fourier Transform	(1) Williams 2009 (CD), (2) Steven Lehrer	ANN:<br>Accuracy: 98.60<br>Sens: 97.80	Adventitious sounds	Bronchovesicular vs Normal bronchial vs Abnormal bronchial vs Crackle vs

			Sound Repository 2002 (CD)	Spec: 100<br><br>ANFIS:<br>Accuracy: 66.40<br>Sens: 56.90<br>Spec: 70.40		Wheeze vs Stridor vs Normal bronchophony vs Bronchophony by consolidation vs Normal egophony vs Abnormal egophony
Palaniappan, 2014 [14]	SVM, k-NN	Mel-Frequency Cepstral Coefficients	R.A.L.E. Lung Sounds	SVM:<br>Accuracy: 92.19<br>Sens: *,<br>Spec: *<br><br>k-NN:<br>Accuracy: 98.26<br>Sens: *<br>Spec: *	Diseases	Norma vs Airway obstruction vs Parenchymal pathology
Paraschiv, 2020 [82]	2D CNN	Mel-Frequency Cepstral Coefficients; Discrete Fourier Transform	ICBHI 2017	Accuracy: 90.21<br>Sens: *<br>Spec: *	Diseases	Bronchiectasis vs Pneumonia vs Bronchiolitis vs COPD vs URTI vs Normal
Petmezas, 2022 [83]	CNN + RNN (LSTM)	Short-time Fourier Transform	ICBHI 2017	Accuracy: 76.39<br>Sens: 52.78<br>Spec: 84.26	Adventitious sounds	Normal vs Crackle vs Wheeze vs Crackle and Wheeze
Pham, 2021 [85]	CNN	Continuous Wavelet Transform	ICBHI 2017	Task 1:<br>Accuracy: *<br>Sens: 32.00<br>Spec: 73.00<br><br>Task 2:<br>Accuracy: *<br>Sens: 85.00<br>Spec: 88.00	Both adventitious sounds and diseases	Task 1: Normal vs Crackle vs Wheeze vs Crackle and Wheeze<br><br>Task 2: Healthy vs Chronic disease (COPD, bronchiectasis, asthma) vs non-Chronic disease (URTI, LRTI, Pneumonia, Bronchiolitis)
Pham, 2021 [84]	C-DNN	Mel-frequency Spectrogram; Gammatone Filterbank Spectrogram; Stacked Mel-Frequency Cepstral Coefficients; Rectangular Constant Q Transform	ICBHI 2017	Task 1.1:<br>Accuracy: *<br>Sens: 68.00<br>Spec: 90.00<br><br>Task 1.2:<br>Accuracy: *<br>Sens: 78.00<br>Spec: 90.00<br><br>Task 2.1:<br>Accuracy: *<br>Sens: 95.00<br>Spec: 86.00<br><br>Task 2.2:	Both adventitious sounds and diseases	Task 1.1: Normal vs Crackle vs Wheeze vs Crackle and Wheeze<br><br>Task 1.2: Normal vs Abnormal<br><br>Task 2.1: Healthy vs Chronic disease (COPD, bronchiectasis, asthma) vs non-Chronic disease (URTI, LRTI, Pneumonia, Bronchiolitis)<br><br>Task 2.2: Healthy vs Unhealthy

				Accuracy: *<br>Sens: 98.00<br>Spec: 86.00		
Pham Thi Viet, 2022 [86]	CNN + Ensemble learning	Continuous Wavelet Transform	ICBHI 2017	Task 1:<br>Accuracy: *<br>Sens: 83.10<br>Spec: 86.40<br><br>Task 2:<br>Accuracy: *<br>Sens: 75.10<br>Spec: 84.20	Adventitious sounds	Task 1: Normal vs Abnormal<br><br>Task 2: Normal vs Crackle vs Wheeze vs Crackle and Wheeze
Porieva, 2021 [107]	QDA	Fast Fourier Transform; Multilevel Wavelet Transform; Mel-Frequency Cepstral Coefficients	CORA database	Accuracy: 93.2<br>Sens: *<br>Spec: *	Diseases	Normal vs Bronchitis vs COPD
Pramono, 2019 [119]	LR	Mel-Frequency Cepstral Coefficients; Tonality Index; Linear Predictive Coding	(1) R.A.L.E. Lung Sounds, (2) Pulmonary Breath Sounds ETSU 2002, (3) 3M Littmann Lung Sounds Library, (4) Stethographics Lung Sound Samples	Accuracy: *<br>Sens: 83.86<br>Spec: 81.19	Adventitious sounds	Normal vs Wheeze
Rocha, 2020 [87]	LDA, SVM, RUSBoost (Type of DT), CNN	Short-term Fourier Transform	ICBHI 2017	LDA:<br>Accuracy: 84.20<br>Sens: *<br>Spec: *<br><br>SVM:<br>Accuracy: 89.70<br>Sens: *<br>Spec: *<br><br>RUSBoost:<br>Accuracy: 92.30<br>Sens: *<br>Spec: *<br><br>CNN:<br>Accuracy: 96.90<br>Sens: *<br>Spec: *	Adventitious sounds	Crackle vs Wheeze vs Others

Sen, 2014 [94]	LIBSVM	Vector Autoregression	Bogazici University Lung Acoustics Laboratory	Task 1:<br>Accuracy: *<br>Sens: 85.00<br>Spec: 85.00<br><br>Task 2:<br>Accuracy: *<br>Sens: 75.00<br>Spec: *	Diseases	Task 1: Healthy vs Pathologic<br><br>Task 2: Healthy vs Bronchiectasis vs Interstitial pulmonary disease
Serbes, 2018 [95]	SVM	Short-time Fourier Transform; Non-linear Resonance Based Wavelet Decomposition	ICBHI 2017	Accuracy: 57.88<br>Sens: 55.29<br>Spec: 83.25	Adventitious sounds	Normal vs crackle vs wheeze vs crackle and wheeze
Shuvo, 2020 [88]	Lightweight CNN	Empirical Mode Decomposition; Continuous Wavelet Transform	ICBHI 2017	Task 1:<br>Accuracy: 98.92<br>Sens: 98.90<br>Spec: 100<br><br>Task 2:<br>Accuracy: 98.70<br>Sens: 98.60<br>Spec: 100	Diseases	Task 1: Healthy vs Chronic disease (COPD, Bronchiectasis, Asthma) vs non-Chronic disease (URTI, LRTI, Pneumonia, Bronchiolitis)<br><br>Task 2: Healthy vs Bronchiectasis vs Bronchiolitis vs COPD vs Pneumonia vs URTI
Sosa, 2015 [97]	SVM	Short-time Fourier Transform; Wavelet Packet Transform; Mel-Frequency Cepstral Coefficients	R.A.L.E. Lung Sounds	Accuracy: *<br>Sens: 81.50<br>Spec: 82.60	Adventitious sounds	Normal vs Wheeze
Stasiakiewicz, 2021 [96]	SVM	Continuous Wavelet Transform	(1) ICBHI 2017,<br>(2) Self-recorded	Accuracy: 98.00<br>Sens: 97.80<br>Spec: 98.20	Diseases	Healthy vs Pneumonia vs Pulmonary fibrosis vs Heart failure vs COPD
Tariq, 2019 [89]	2D CNN	Mel-frequency Spectrogram	ICBHI 2017	Accuracy: 97.00<br>Sens: *<br>Spec: *	Diseases	Healthy vs Asthma vs COPD vs Bronchiectasis vs Pneumonia vs LRTI vs URTI
Tasar, 2022 [98]	k-NN, SVM, DT	Tunable Q Factor Wavelet; Iterative Neighborhood	ICBHI 2017	Task 1:<br><br>k-NN:<br>Accuracy: 99.45<br>Sens: 99.52<br>Spec: 98.78	Diseases	Task 1: Healthy vs Bronchiolitis vs COPD vs Bronchiectasis vs Pneumonia vs LRTI vs URTI<br><br>Task 2: COPD vs Pneumonia vs Asthma

		Component Analysis		SVM:<br>Accuracy: 98.41<br>Sens: 96.97<br>Spec: 99.01<br><br>DT:<br>Accuracy: 83.70<br>Sens: 77.76<br>Spec: 78.91<br><br>Task 2:<br><br>k-NN:<br>Accuracy: 99.31<br>Sens: 99.57<br>Spec: 99.46<br><br>SVM:<br>Accuracy: 97.87<br>Sens: 98.55<br>Spec: 98.48<br><br>DT:<br>Accuracy: 96.66<br>Sens: 94.35<br>Spec: 97.19<br><br>Task 3:<br><br>k-NN:<br>Accuracy: 99.19<br>Sens: 99.01<br>Spec: 98.77<br><br>SVM:<br>Accuracy: 97.80<br>Sens: 96.63<br>Spec: 98.19<br><br>DT:<br>Accuracy: 86.41<br>Sens: 82.06<br>Spec: 82.40		Task 3: Healthy vs Asthma vs COPD vs Bronchiectasis vs Bronchiolitis vs Pneumonia vs LRTI vs URTI
Tocchetto, 2014 [36]	ANN	Wavelet Packet Transform	Steven Lehrer Sound Repository (CD)	Accuracy: 99.26<br>Sens: *<br>Spec: *	Adventitious sounds	Normal vs Wheeze vs Crackle
Tripathy, 2022 [117]	LGBM	Empirical Wavelet Transform	KAUH database	Accuracy: 84.76<br>Sens: 91.12<br>Spec: 82.96	Diseases	Healthy vs asthma vs COPD vs Pneumonia

Vidhya, 2022 [99]	GB, SVM, k-NN	Mel-Frequency Cepstral Coefficients	ICBHI 2017	GB:<br>Accuracy: 97.00<br>Sens: *<br>Spec: *<br><br>SVM:<br>Accuracy: 69.40<br>Sens: *<br>Spec: *<br><br>k-NN:<br>Accuracy: 90.23<br>Sens: *<br>Spec: *	Diseases	Healthy vs Pneumonia
Yang, 2020 [90]	CNN (ResNet)	Short-time Fourier Transform	ICBHI 2017	Accuracy: *<br>Sens: 17.80<br>Spec: 81.20	Adventitious sounds	Normal vs Crackle vs Wheeze vs Crackle and Wheeze
Yi Ma, 2019 [91]	Bi-ResNet NN	Short-time Fourier Transform; Wavelet Packet Transform	ICBHI 2017	Accuracy: 67.44<br>Sens: 58.54<br>Spec: 80.06	Adventitious sounds	Normal vs Crackle vs Wheeze vs Crackle and Wheeze
Zulfiqar, 2021 [18]	AlexNet, InceptionNet, ResNet	Fourier Transform	(1) R.A.L.E. Lung Sounds, (2) Thinklabs database, (3) Easy Auscultation database (4) Others	AlexNet:<br>Accuracy: 100<br>Sens: 100<br>Spec: *<br><br>InceptionNet:<br>Accuracy: 95.00<br>Sens: 95.00<br>Spec: *<br><br>ResNet:<br>Accuracy: 95.00<br>Sens: 93.00<br>Spec: *<br><br>LeNet:<br>Accuracy: 89.00<br>Sens: 90.00<br>Spec: *	Adventitious sounds	Coarse crackle vs. Fine crackle vs. Pleural rub vs. Rhonchi vs. Squawk vs. Stridor vs. Wheeze

Legend: ANFIS: Adaptive Neuro-Fuzzy Interference Systems; BDLSTM: Bi-Directional Long Short-Term Memory Neural Network; DAG: Directed Acyclic Graph (type of CNN); DBN: Deep Belief Networks; DNN: Deep neural network; DS-CNN: Depthwise Separable Convolutional Neural Network; DT: Decision Tree; ELM: Extreme Learning Machine; GMM: Gaussian Mixture Models; HMM: Hidden Markov Model; k-NN: k-Nearest Neighbor; LDA: Linear Discriminant Analysis; LGBM: Light Gradient Boosting Machine; LR: Logistic Regression; MLP: Multi-Layer Perceptron; NM-RNN: Noise-Masking Recurrent Neural Network; QD: Quadratic Discriminant Analysis (type of Discriminant Analysis); RF: Random Forest; RSE: Random Subspace Ensembles; RUSBoost: Random Undersampling Boosted Trees; SE: Snapshot Ensemble (type of CNN); SVM: Support Vector Machine; XSG: Xilinx System Generator

*Not provided in the manuscript.

Table S2. Individual risk of bias assessment. Sorted in the same order as Table S1.

**Author, Year**	**Patient Selection**	**Index Test**	**Reference Standard**	**Flow and timing**
Acharya, 2020 [57]	High	Low	High	Low
Alqudah, 2022 [58]	High	Low	High	Low
Altan, 2020 [59]	High	Low	High	Low
Bahoura, 2009 [35]	High	Low	High	Unclear
Bahoura, 2018 [60]	High	Low	High	Unclear
Bardou, 2018 [61]	Unclear	Low	High	Unclear
Basu, 2020 [62]	High	Low	High	Low
Boujelben, 2018 [93]	High	Low	High	Unclear
Brunese, 2022 [63]	High	Low	High	Low
Chen, 2019 [64]	High	Low	High	Low
Chen, 2019 [65]	High	Low	High	Unclear
Datta, 2017 [37]	High	Low	High	High
Demir, 2020 [67]	High	Low	High	Low
Demir, 2020 [66]	High	Low	High	Low
D. Perna, 2018 [68]	High	Low	High	Low
Fraiwan, 2022 [69]	High	Low	High	Low
Gairola, 2017 [70]	High	Low	High	Low
Garcia-Ordas, 2020 [71]	High	Low	High	Low
G. Chambres, 2018 [102]	High	Low	High	Low
Hazra, 2020 [72]	High	Low	High	Low
Jaber, 2020 [109]	Unclear	Low	High	High
Jakovljevic, 2018 [113]	High	Low	High	Low
Jung, 2021 [73]	Unclear	Low	High	Low
Kochetov, 2018 [74]	High	Low	High	Low
Kok, 2019 [103]	High	Low	High	Low
Li, 2022 [75]	High	Low	High	Low
Li, 2021 [76]	High	Low	High	Low
Lu, 2008 [34]	High	Low	High	High
Minami, 2019 [77]	High	Low	High	Low
Monaco, 2020 [78]	High	Low	High	Low
Mukherjee, 2021 [79]	High	Low	High	Low
Naqvi, 2020 [106]	High	Low	High	Low
Naves, 2016 [39]	Unclear	Low	Unclear	Unclear

Ngo, 2021 [80]	High	Low	High	Low
Nguyen, 2020 [81]	High	Low	High	Low
Ntalampiras, 2020 [114]	High	Low	High	Low
Ntalampiras, 2020 [111]	High	Low	High	Low
Oletic, 2014 [104]	High	Low	High	High
Oletic, 2018 [115]	High	Low	High	High
Oweis, 2015 [38]	High	Low	Unclear	Unclear
Palaniappan, 2014 [14]	Unclear	Low	High	Unclear
Paraschiv, 2020 [82]	High	Low	High	Low
Petmezas, 2022 [83]	High	Low	High	Low
Pham, 2021 [85]	High	Low	High	Low
Pham, 2021 [84]	High	Low	High	Low
Pham Thi Viet, 2022 [86]	High	Low	High	Low
Porieva, 2021 [107]	Unclear	Low	Unclear	Unclear
Pramono, 2019 [119]	High	Low	High	Unclear
Rocha, 2020 [87]	High	Low	High	Low
Sen, 2014 [94]	Unclear	Low	High	High
Serbes, 2018 [95]	High	Low	High	Low
Shuvo, 2020 [88]	High	Low	High	High
Sosa, 2015 [97]	Unclear	Low	High	Unclear
Stasiakiewicz, 2021 [96]	High	Low	High	High
Tariq, 2019 [89]	High	Low	High	Low
Tasar, 2022 [98]	High	Low	High	Low
Tocchetto, 2014 [36]	Unclear	Low	Unclear	Unclear
Tripathy, 2022 [117]	Low	Low	High	Low
Vidhya, 2022 [99]	High	Low	High	Low
Yang, 2020 [90]	High	Low	High	Low
Yi Ma, 2019 [91]	High	Low	High	Low
Zulfiqar, 2021 [18]	High	Low	High	High

Table S3. Individual applicability concerns assessment. Sorted in the same order as Table S1.

Author, Year	Patient Selection	Index Test	Reference Standard
Acharya, 2020 [57]	High	Low	High
Alqudah, 2022 [58]	High	Low	High
Altan, 2020 [59]	Low	Low	High
Bahoura, 2009 [35]	Uncertain	Low	High
Bahoura, 2018 [60]	Uncertain	Low	High
Bardou, 2018 [61]	Uncertain	Low	High

Basu, 2020 [62]	High	Low	High
Boujelben, 2018 [93]	Uncertain	Low	High
Brunese, 2022 [63]	High	Low	High
Chen, 2019 [64]	High	Low	High
Chen, 2019 [65]	High	Low	High
Datta, 2017 [37]	Uncertain	Low	High
Demir, 2020 [67]	High	Low	High
Demir, 2020 [66]	High	Low	High
D. Perna, 2018 [68]	High	Low	High
Fraiwan, 2022 [69]	High	Low	High
Gairola, 2017 [70]	High	Low	High
Garcia-Ordas, 2020 [71]	High	Low	High
G. Chambres, 2018 [102]	High	Low	High
Hazra, 2020 [72]	High	Low	High
Jaber, 2020 [109]	Uncertain	Low	High
Jakovljevic, 2018 [113]	High	Low	High
Jung, 2021 [73]	Low	Low	High
Kochetov, 2018 [74]	High	Low	High
Kok, 2019 [103]	High	Low	High
Li, 2022 [75]	High	Low	High
Li, 2021 [76]	High	Low	High
Lu, 2008 [34]	Uncertain	Low	High
Minami, 2019 [77]	High	Low	High
Monaco, 2020 [78]	High	Low	High
Mukherjee, 2021 [79]	High	Low	High
Naqvi, 2020 [106]	High	Low	High
Naves, 2016 [39]	Uncertain	Low	High
Ngo, 2021 [80]	High	Low	High
Nguyen, 2020 [81]	High	Low	High
Ntalampiras, 2020 [114]	High	Low	High
Ntalampiras, 2020 [111]	High	Low	High
Oletic, 2014 [104]	Uncertain	Low	High
Oletic, 2018 [115]	Uncertain	Low	High
Oweis, 2015 [38]	Uncertain	Low	High
Palaniappan, 2014 [14]	Uncertain	Low	High
Paraschiv, 2020 [82]	High	Low	High
Petmezas, 2022 [83]	High	Low	High
Pham, 2021 [85]	High	Low	High
Pham, 2021 [84]	High	Low	High
Pham Thi Viet, 2022 [86]	High	Low	High
Porieva, 2021 [107]	Uncertain	Low	High
Pramono, 2019 [119]	Uncertain	Low	High

Rocha, 2020 [87]	High	Low	High
Sen, 2014 [94]	Uncertain	Low	High
Serbes, 2018 [95]	High	Low	High
Shuvo, 2020 [88]	High	Low	High
Sosa, 2015 [97]	Uncertain	Low	High
Stasiakiewicz, 2021 [96]	High	Low	High
Tariq, 2019 [89]	High	Low	High
Tasar, 2022 [98]	High	Low	High
Tocchetto, 2014 [36]	Uncertain	Low	High
Tripathy, 2022 [117]	High	Low	High
Vidhya, 2022 [99]	High	Low	High
Yang, 2020 [90]	High	Low	High
Yi Ma, 2019 [91]	High	Low	High
Zulfiqar, 2021 [18]	Uncertain	Low	High

Search strategy:

Cochrane Central Register of Controlled Trials (CCTR) via Ovid (1991+)):

(((auscultat* or stethoscope* or ((lung* or breath* or respirat* or pulmonary) adj2 sound*)).ab,hw,ti. AND (remote* or tele* or bio-tele* or ehealth or e-health or mhealth or m-health or computeri* or digit* or electr* or wireless* or visual* or automat* or smartphone* or smart-phone* or mobile-app* or virtual*).ab,hw,ti. AND ((remote* or tele* or biotele* or ehealth or e-health or mhealth or m-health or computeri* or digit* or electr* or wireless* or visual* or automat* or smartphone* or phone* or mobile-app* or virtual*).ti,kw. or ((auscultat* or stethoscope* or ((lung* or breath* or respirat* or pulmonary) adj2 sound*)) adj4 (remote* or tele* or biotele* or ehealth or e-health or computeri* or digit* or electr* or wireless* or visual* or automat* or smartphone* or phone* or mobile-app* or virtual*)).ab,kw,ti.) or (teleauscultat* or digiscope*).ab,hw,ti.) AND (airway or asthma* or bronch* or cardiopulmo-nary or chest or COPD or diaphragm or lung* or mediastinal or pleur* or pneumo* or pulmonary or thora* or trach*).ab,hw,ti.) NOT ((canine* or dog or dogs or feline* or hamster* or lamb or lambs or sheep or mice or mouse or murine or monkey* or pig* or porcine or primate* or rabbit* or rat or rats or rodent* or horse* or equine* or veterinar*).ti,hw. OR (fetal or fetus or newborn* or neonat* or infant* or toddler* or child* or adolesc* or teen* or youth or school* or pediatric* or paediatric*).ti.)

Embase via Ovid (1974+):

((exp auscultation/ or exp abnormal respiratory sound/ or (auscultat* or stethoscope* or ((lung* or breath* or respirat* or pulmo-nary) adj2 sound*)).ab,kw,ti.) AND (exp telecommunication/ or videoconferencing/ or wireless communication/ or exp telemetry/ or exp amplifier/ or smartphone/ or exp mobile application/ or (remote* or tele* or biotele* or ehealth or e-health or mhealth or m-health or computeri* or digit* or electr* or wireless* or visual* or automat* or smartphone* or smart-phone* or mobile-app* or vir-tual*).ab,kw,ti.) AND (exp *telecommunication/ or *videoconferencing/ or *wireless communication/ or exp *telemetry/ or *smartphone/ or exp *mobile application/ or exp *amplifier/ or exp *auscultation/ or (remote* or tele* or biotele* or ehealth or e-health or mhealth or m-health or computeri* or digit* or electr* or wireless* or visual* or automat* or smartphone* or phone* or mobile-app* or virtual*).ti,kw. or ((auscultat* or stethoscope* or ((lung* or breath* or respirat* or pulmonary) adj2 sound*)) adj4 (remote* or tele* or biotele* or ehealth or e-health or computeri* or digit* or electr* or wireless* or visual* or automat* or smartphone* or phone* or mobile-app* or virtual*)).ab,kw,ti,tw. or (teleauscultat* or digiscope*).ab,kw,ti.) AND (exp respiratory

tract disease/ or exp respiratory tract disease assessment/ or (airway or asthma* or bronch* or cardiopulmonary or chest or COPD or diaphragm or lung* or mediastinal or pleur* or pneumo* or pulmonary or thora* or trach*).ab,kw,ti,tw.)) NOT ((exp animal/ or exp juvenile animal/ or adult animal/ or animal cell/ or animal tissue/ or nonhuman/ or animal experiment/ or animal model/) not exp human/ or (canine* or dog or dogs or feline* or hamster* or lamb or lambs or sheep or mice or mouse or murine or monkey* or pig* or porcine or primate* or rabbit* or rat or rats or rodent* or horse* or equine* or veterinar*).ti,kw,dq,jx. OR (exp juvenile/ not adult/)) Limit to English, 1990+

MEDLINE via Ovid (1946+ and Epub Ahead of Print, In-Process & Other Non-Indexed Citations and Ovid MEDLINE(R) Daily):

((exp Auscultation/ or Respiratory Sounds/ or (auscultat* or stethoscope* or ((lung* or breath* or respirat* or pulmonary) adj2 sound*)).ab,kf,ti.) AND (exp Telecommunications/ or Amplifiers, Electronic/ or Smartphone/ or Mobile Applications/ or (remote* or tele* or biotele* or ehealth or e-health or computeri* or digit* or electr* or wireless* or visual* or automat* or smartphone* or smart-phone* or mobile-app* or virtual*).ab,kw,ti.) AND (exp *Telecommunications/ or *Amplifiers, Electronic/ or *Smartphone/ or *Mobile Applications/ or (remote* or tele* or biotele* or ehealth or e-health or mhealth or m-health or computeri* or digit* or electr* or wireless* or visual* or automat* or smartphone* or phone* or mobile-app* or virtual*).ti,kw. or ((auscultat* or stethoscope* or ((lung* or breath* or respirat* or pulmonary) adj2 sound*)) adj4 (remote* or tele* or biotele* or ehealth or e-health or mhealth or m-health or computeri* or digit* or electr* or wireless* or visual* or automat* or smartphone* or phone* or mobile-app* or virtual*)).ab,kf,ti,tw. or (teleauscultat* or digiscope*).ab,kw,ti.) AND (exp Respiratory Tract Diseases/ or (airway or asthma* or bronch* or cardiopulmonary or chest or COPD or diaphragm or lung* or mediastinal or pleur* or pneumo* or pulmonary or thora* or trach*).ab,kw,ti,tw.)) NOT ((exp Animals/ or Models, Animal/ or Disease Models, Animal/) not Humans/ or (canine* or dog or dogs or feline* or hamster* or lamb or lambs or sheep or mice or mouse or murine or monkey* or pig* or porcine or primate* or rabbit* or rat or rats or rodent* or horse* or equine* or veterinar*).ti,kw,jw. OR (exp Infant/ or exp CHILD/) not exp ADULT/)) Limit to English, 1990+

Scopus via Elsevier (1970+):

(((TITLE-ABS-KEY (auscultat* OR stethoscope*) OR TITLE-ABS-KEY ((lung* OR breath* OR respirat* OR pulmonary) W/2 sound*))) AND (TITLE-ABS-KEY (remote* OR tele* OR biotele* OR ehealth OR e-health OR mhealth OR m-health OR computeri* OR digit* OR electr* OR wireless* OR visual* OR automat* OR smartphone* OR smart-phone* OR mobile-app* OR virtual*)) AND ((TITLE (remote* OR tele* OR biotele* OR ehealth OR e-health OR mhealth OR m-health OR computeri* OR digit* OR electr* OR wireless* OR visual* OR automat* OR smartphone* OR phone* OR mobile-app* OR virtual*) OR KEY (remote* OR tele* OR biotele* OR ehealth OR e-health OR mhealth OR m-health OR computeri* OR digit* OR electr* OR wireless* OR visual* OR automat* OR smartphone* OR phone* OR mobile-app* OR virtual*) OR TITLE-ABS-KEY (teleauscultat* OR digiscope*) OR TITLE-ABS-KEY ((auscultat* OR stethoscope* OR ((lung* OR breath* OR respirat* OR pulmonary) W/2 sound*)) W/4 (remote* OR tele* OR biotele* OR ehealth OR e-health OR computeri* OR digit* OR electr* OR wireless* OR visual* OR automat* OR smartphone* OR phone* OR mobile-app* OR virtual*)))) AND (TITLE-ABS-KEY (airway OR asthma* OR bronch* OR cardiopulmonary OR chest OR copd OR diaphragm OR lung* OR mediastinal OR pleur* OR pneumo* OR pulmonary OR thora* OR trach*))) AND NOT (TITLE (canine* OR dog OR dogs OR feline* OR hamster* OR lamb OR lambs OR sheep OR mice OR mouse OR murine OR monkey* OR pig* OR porcine OR primate* OR rabbit* OR rat OR rats OR rodent* OR horse* OR equine* OR veterinar* OR fetal OR fetus OR newborn* OR neonat* OR infant* OR toddler* OR child* OR adolesc* OR teen* OR youth OR school* OR pediatric* OR paediatric*) OR SUBJAREA (vete)) AND (LIMIT-TO (SRCTYPE,"j")) AND (LIMIT-TO (DOCTYPE,"ar") OR

LIMIT-TO (DOCTYPE,"cp") OR LIMIT-TO (DOCTYPE,"re") OR LIMIT-TO (DOCTYPE,"le") OR LIMIT-TO (DOCTYPE,"no") OR LIMIT-TO (DOCTYPE,"sh") OR LIMIT-TO (DOCTYPE,"ed") OR LIMIT-TO (DOCTYPE,"dp")) AND (LIMIT-TO (PUBYEAR,2021) OR LIMIT-TO (PUBYEAR,2020) OR LIMIT-TO (PUBYEAR,2019) OR LIMIT-TO (PUBYEAR,2018) OR LIMIT-TO (PUBYEAR,2017) OR LIMIT-TO (PUBYEAR,2016) OR LIMIT-TO (PUBYEAR,2015) OR LIMIT-TO (PUBYEAR,2014) OR LIMIT-TO (PUBYEAR,2013) OR LIMIT-TO (PUBYEAR,2012) OR LIMIT-TO (PUBYEAR,2011) OR LIMIT-TO (PUBYEAR,2010) OR LIMIT-TO (PUBYEAR,2009) OR LIMIT-TO (PUBYEAR,2008) OR LIMIT-TO (PUBYEAR,2007) OR LIMIT-TO (PUBYEAR,2006) OR LIMIT-TO (PUBYEAR,2005) OR LIMIT-TO (PUBYEAR,2004) OR LIMIT-TO (PUBYEAR,2003) OR LIMIT-TO (PUBYEAR,2002) OR LIMIT-TO (PUBYEAR,2001) OR LIMIT-TO (PUBYEAR,2000) OR LIMIT-TO (PUBYEAR,1999) OR LIMIT-TO (PUBYEAR,1998) OR LIMIT-TO (PUBYEAR,1997) OR LIMIT-TO (PUBYEAR,1996) OR LIMIT-TO (PUBYEAR,1995) OR LIMIT-TO (PUBYEAR,1994) OR LIMIT-TO (PUBYEAR,1993) OR LIMIT-TO (PUBYEAR,1992) OR LIMIT-TO (PUBYEAR,1991) OR LIMIT-TO (PUBYEAR,1990)) AND (LIMIT-TO (LANGUAGE,"English")) AND (LIMIT-TO (EXACTKEYWORD,"Lung Auscultation") OR LIMIT-TO (EXACTKEYWORD,"Auscultation"))

Web of Science Core Collection via Clarivate Analytics (1975+):

((TS=(auscultat* or stethoscope* or ((lung* or breath* or respirat* or pulmonary) NEAR/2 sound*)) AND TS=(remote* or tele* or biotele* or ehealth or e-health or mhealth or m-health or computeri* or digit* or electr* or wireless* or visual* or automat* or smartphone* or smart-phone* or mobile-app* or virtual*) AND (TITLE=(remote* or tele* or biotele* or ehealth or e-health or mhealth or m-health or computeri* or digit* or electr* or wireless* or visual* or automat* or smartphone* or phone* or mobile-app* or virtual*) OR TS=((auscultat* or stethoscope* or lung-sound* or breath-sound* or respiratory-sound* or respiration-sound* or pulmonary-sound*) NEAR/4 (remote* or tele* or biotele* or ehealth or e-health or computeri* or digit* or electr* or wireless* or visual* or automat* or smartphone* or phone* or mobile-app* or virtual*)) OR TS=breathing-sound*) OR TS=(teleauscultat* or digiscope*) AND (TS=(airway or asthma* or bronch* or cardiopulmonary or chest or COPD or diaphragm or lung* or mediastinal or pleur* or pneumo* or pulmonary or thora* or trach*)) NOT (TI=(canine* or dog or dogs or feline* or hamster* or lamb or lambs or sheep or mice or mouse or murine or monkey* or pig* or porcine or primate* or rabbit* or rat or rats or rodent* or horse* or equine* or veterinar* or fetal or fetus or newborn* or neonat* or infant* or toddler* or child* or adolesc* or teen* or youth or school* or pediatric* or paediatric*) OR SU=veterin) Limit to English, 1990+, exclude meeting abstracts, book chapters
